# Supplementary material for: Understanding the challenges of healthcare transition in the context of HIV-related stigma for young adults with perinatal HIV in Thailand
Source: PLoS One. 2025 Nov 26;20(11):e0336624. doi: 10.1371/journal.pone.0336624 (PMC12654928; doi:10.1371/journal.pone.0336624)
Supplement: Attachment — Submitted filename: Responses to reviewers.docx [file pone.0336624.s003.docx]

**Response to reviewers’ comments**

 Journal requirements:

When submitting your revision, we need you to address these additional requirements.

1. Please ensure that your manuscript meets PLOS ONE's style requirements, including those for file naming. The PLOS ONE style templates can be found at

[https://journals.plos.org/plosone/s/file?id=wjVg/PLOSOne_formatting_sample_main_body.pdf](https://track.editorialmanager.com/CL0/https:%2F%2Fjournals.plos.org%2Fplosone%2Fs%2Ffile%3Fid=wjVg%2FPLOSOne_formatting_sample_main_body.pdf/1/010f0196ae8cbc12-40da9c15-c92d-409c-8dd7-34bdfcfa0a1b-000000/yxF8mor5qyohocDk9GnLZOOeqqZkGqEoTh7ONGE7RZQ=210)  and

[https://journals.plos.org/plosone/s/file?id=ba62/PLOSOne_formatting_sample_title_authors_affiliations.pdf](https://track.editorialmanager.com/CL0/https:%2F%2Fjournals.plos.org%2Fplosone%2Fs%2Ffile%3Fid=ba62%2FPLOSOne_formatting_sample_title_authors_affiliations.pdf/1/010f0196ae8cbc12-40da9c15-c92d-409c-8dd7-34bdfcfa0a1b-000000/YJSnd8WZrh87KWDqVKbSTC4-Abrc5ZjKWYR_jijDcVE=210)

**Response:** We have rechecked the manuscript style.

2. Thank you for stating the following financial disclosure:

“This study was supported by grants to amfAR from ViiV Healthcare, and the U.S. National Institutes of Health’s National Institute of Allergy and Infectious Diseases, the Eunice Kennedy Shriver National Institute of Child Health and Human Development, the National Cancer Institute, the National Institute of Mental Health, the National Institute on Drug Abuse, the National Heart, Lung, and Blood Institute, the National Institute on Alcohol Abuse and Alcoholism, the National Institute of Diabetes and Digestive and Kidney Diseases, and the Fogarty International Center, as part of the International Epidemiology Databases to Evaluate AIDS (IeDEA; U01AI069907).”

Please state what role the funders took in the study.  If the funders had no role, please state: "The funders had no role in study design, data collection and analysis, decision to publish, or preparation of the manuscript."

If this statement is not correct you must amend it as needed.

Please include this amended Role of Funder statement in your cover letter; we will change the online submission form on your behalf.

**Response:** We have included this change in the cover letter.

3. We note that you have indicated that there are restrictions to data sharing for this study. For studies involving human research participant data or other sensitive data, we encourage authors to share de-identified or anonymized data. However, when data cannot be publicly shared for ethical reasons, we allow authors to make their data sets available upon request. For information on unacceptable data access restrictions, please see <http://journals.plos.org/plosone/s/data-availability#loc-unacceptable-data-access-restrictions>.

Before we proceed with your manuscript, please address the following prompts:

a) If there are ethical or legal restrictions on sharing a de-identified data set, please explain them in detail (e.g., data contain potentially identifying or sensitive patient information, data are owned by a third-party organization, etc.) and who has imposed them (e.g., a Research Ethics Committee or Institutional Review Board, etc.). Please also provide contact information for a data access committee, ethics committee, or other institutional body to which data requests may be sent.

b) If there are no restrictions, please upload the minimal anonymized data set necessary to replicate your study findings to a stable, public repository and provide us with the relevant URLs, DOIs, or accession numbers. Please see <http://www.bmj.com/content/340/bmj.c181.long> for guidelines on how to de-identify and prepare clinical data for publication. For a list of recommended repositories, please see <https://journals.plos.org/plosone/s/recommended-repositories>. You also have the option of uploading the data as Supporting Information files, but we would recommend depositing data directly to a data repository if possible.

Please update your Data Availability statement in the submission form accordingly.

**Response:** The data contains potentially identifying and sensitive patient information. We would choose to state that the data sets available upon request. This will be updated in the submission form during resubmitting.

Additional Editor Comments:

1. Clarity and Framing of Findings in Light of Sample Size
The reviewer highlighted concerns regarding the presentation of quantitative figures within a primarily qualitative study (e.g., reporting percentages such as “60% had low levels of stigma”). Given the small sample size (n=20), such statistics can be misleading and may imply a level of precision not supported by qualitative methodology. We recommend rephrasing these statements to reflect qualitative trends or patterns without numeric quantification unless these are triangulated with validated scales.

**Response:** We revised the results in this part. It is now read as follows.

*“The Thai IHSS brief score revealed a low level of internalized stigma in the study participants (median score 14; IQR 11-17). The components with the highest scores indicating their most concern were anticipated negative thoughts (Items 1 & 2) and negative thoughts toward self (Items 5 & 6).”*

We also removed the numbers in the abstracts and replaced them with the following sentence.

*“The Thai IHSS brief score revealed a low level of internalized stigma in the study participants (median score 14; IQR 11-17).”*

2. Interpretation and Generalizability
The reviewer has pointed out that comparisons with studies involving larger sample sizes (e.g., references 16 and 17 with >90 participants) should be made cautiously. Please consider adding a brief reflection on how the limited sample size may affect the transferability of findings and how these limitations were managed methodologically and interpretatively.

**Response:** We revised the 2^nd^ and 3^rd^ paragraphs of the discussion as follows.

*“A larger study among 93 Nigerian youth with HIV (median age of 19.5 years) reported the presence of internalized stigma in 62% [16]. They found more negative self-image (stereotypes about HIV) in the younger age group (10-19 years), and more personalized stigma in the older age group (20-26 years). In our study, we enrolled selected participants who were willing to talk and share experiences; at the median age of 23 years, both components of internalized stigma were presented. However, the low level of internalized stigma in this study might not represent the larger group of YPHIV in the country.*

*A US study gathered data from a survey in 95 PLWH described that internalized or self-stigma occurs when one absorbs negative messages and stereotypes about HIV, comes to personalize them, and apply to themselves [17]. The absorption and personalization of HIV-related messages to themselves were applied to our study participants. As they acquired HIV from vertical transmission, having HIV was not related to their health behaviors.”*

3. Limitations Section
It is very important to explicitly highlight the limitations of the study alongside its strengths. Doing so will not only help contextualize the findings, especially those that may appear limited due to sample size or scope, but also provide a more comprehensive understanding of the study's contribution. We encourage you to reflect on methodological constraints, such as sample size and participant selection, and to discuss how these may impact the transferability or generalizability of the results.

**Response:** We added the study strength and limitation as follows.

*“Our study was among a few qualitative studies in this region focusing on healthcare transition. We revealed the influence of HIV-related stigma on the transition journey of YPHIV, which provided a comprehensive understanding. The study limitation included selection bias, as we selected participants willing to join, which might represent the group with more positive experiences than those who did not show interest in participating. Although we conducted a multiple-method study and collected some quantitative data, the estimated sample size was to achieve qualitative data saturation and was probably too few to draw a conclusion or generalize to other young people living with HIV.*”

**Review Comments to the Author**
Reviewer #1: This is an interesting article and fairly well writ ten. However it may benefit from a tighter and shorter draft. Otherwise may I commend for a work well done. May benfit from a review of the English. congrats

Reviewer #2: I want to appreciate the authors for putting together this paper and highlighting an important but a neglected part of HIV care services.

Title and the abstract
1) The title is not very descriptive. Please see two suggestions:
o Understanding the challenges of healthcare transition in the context of HIV-related stigma for young adults with perinatal HIV in Thailand.
o A study of HIV-related stigma and its influence on the healthcare transition journeys of young individuals with perinatal HIV in Thailand

**Response:** Thank you for the proposed titles. We would rather go with the first one.

In the revised manuscript, the title was changed as follows.

*“Understanding the challenges of healthcare transition in the context of HIV-related stigma for young adults with perinatal HIV in Thailand.”*

• Overall, the abstract needs clarity.
2) This statement (with numbers) cannot be part of the result of a qualitative study: Twelve YPHIV (60%) had low, six (30%) had medium, and two (10%) had high levels of internalized stigma. Please rephrase.

**Response:** We have replaced the statement with the following sentence.

*“The median Thai IHSS brief score was 14 (IQR 11-17) representing a low level of internalized stigma in the overall study participants.”*

• If there were two or three groups, there is no mention of the findings from the interaction with care givers.

• Revise the result and start each finding by clearly mentioning which group it refers to.

**Response:** The qualitative findings were from YPHIV in group A, B, and caregivers in group C. We revised the results of abstracts to make it clearer as follows.

*“HIV-related stigma experiences of YPHIV and caregivers were grouped into 3 themes: internalized, anticipated, and enacted stigma/discrimination. Transition experiences of YPHIV in both groups included hesitation to navigate care in adult clinics and feeling unprepared, perceived loss of support, and demotivation from being in care. Anticipated stigma and social problems were expressed by YPHIV and caregivers.”*

• The conclusion section of the abstract should contain one or one and a half sentences to summarize the findings, and then it should end with a recommendation. Please revise.

**Response:** We revised the conclusion of the abstract as follows.

*“In conclusion, we found many stigma issues started since childhood, plus collective experience while growing up. The internalized HIV-related stigma influenced the healthcare transition journey of YPHIV. Healthcare providers need additional guidance on how to manage transition in YPHIV, including* *individualized transition plans for those at increased risk of adverse outcomes, interventions to manage internalized stigma, and follow-up strategies after transition.”*

• Is developing “individualized transitional plans” practically possible, as mentioned in the concluding sentence?

**Response:** Yes, it is possible for selected cases, but not for every case. We revised it to be more realistic as follows.

*“Individualized transition plans for those at increased risk of adverse outcomes,…”*

Introduction
• In the last paragraph of the introduction, please write YPHIV in full.

**Response:** We added full term. It is now read as follows.

*“young people with perinatal HIV (YPHIV)”*

Methods
• What is a multiple-method study?

**Response:** Multiple-methods study or multimethod study is a study using two or more methods in data collection in parallel but does not integrate them in any stages until analysis. We refer to the definition in the reference below.

https://www.researchgate.net/publication/235413072_Toward_a_Definition_of_Mixed_Methods_Research_Journal_of_Mixed_Methods_Research_1_112-133

• Was the FGD done with each of the groups?

**Response:** No, they were mixed. We revised the last sentence in the Study settings and Population to clarify this as follows.

*“We invited participants who underwent IDIs in either Group A or B who were interested in participating on another separate day by appointment.”*

• No description of the Thai Internalized HIV related Stigma Scale brief (Thai-IHSS brief)

**Response:** We added descriptions of IHSS in the Methods section as follows.

*“The 8-item Thai Internalized HIV-related Stigma Scale brief (Thai-IHSS brief) was used to assess internalized stigma. It is a tool developed from survey data among Thai PLWH and had a congruence validity and discriminant power in measuring internalized stigma [14].”*

• Mention inclusion and exclusion criteria; clearly mention that the participants were positive because of vertical transmission

**Response:** We added a phrase to indicate vertical transmission as suggested. It is now read as follows.

*“We purposely recruited YPHIV aged between 18-30 years with a known history of vertical transmission…”*

• Mention more about how participants were approached and recruited

**Response:** We have added details about approaching and recruitment in the Methods section as follows.

*“Potential participants were approached by study staff in the clinics during their regular visit for HIV care or reached out by phone for those who had transitioned out. Those who were interested underwent informed consent process before study enrollment.”*

• What is PHQ-9 scores?

**Response:** We have added the full term of PHQ-9 which is a depression screening tool routinely used in both study clinics, and the scores were obtained from medical records. It is read as follows.

*“The Patient Health Questionnaire (PHQ-9) scores for depression obtained from medical records.”*

**Results**
• With such a small sample size, there is limited justification for having Tables 1 and 2.

**Response:** We revised the manuscript and decided to keep Table 1 to provide the characteristics of study participants, while removing Table 2 due to small sample size as suggested.

• The two studies mentioned in the discussion (references 16 and 17) had a sample size of 90 plus.

**Response:** We understand that our study, which was mainly qualitative, was far too small for comparison. However, we mentioned the similarity of findings (they went in the same direction) and referred to what was previously described in their studies that was applicable to ours.

• The 17% depressive symptom statement should be deleted simply because the sample size is too small and also because the study is primarily a qualitative study.

**Response:** We deleted the percentage of depression from the text Results. However, depression was considered among characteristics of study participants. Thus, we presented it in Table 1.

• Lines 146-151 on printed page 9 should be deleted.

**Response:** Level of HIV-related stigma is a variable of interest in this study. We know that the sample size was small, but the findings is important to fulfill the whole picture of the study. We revised to make it more qualitative as follows.

“The median Thai IHSS brief score was 14 (IQR 11-17) representing a low level of internalized stigma in the overall study participants. The components with the highest scores indicating their most concern were anticipated negative thoughts (“Others may end their relationship with me if they learn that I am living with HIV”, and “Others will think it serves me right if they know I am living with HIV”) and negative thoughts toward self (“I am ashamed that I am living with HIV” and “I think that I have HIV because of my bad karma”).”

• When quoting and describing the quote, clearly mention which group it is coming from.
**Response:** We revised the manuscript and specified the group on each quote. Please see an example as follows.

“[Group A: C-03, male]”

Discussion
• The overall discussion is well written, covering critical areas of the lives of young people with perinatal HIV.
• Please write YPHV in full in the opening paragraph.

**Response:** We added the full term of YPHIV in the first sentence of the discussion as follows.

*“We found that more than half of young people living with HIV (YPHIV)…”*

• Please add a few sentences about the limitations of the study

**Response:** We added the study strength and limitation as follows.

*“Our study was among a few qualitative studies in this region focusing on healthcare transition. We revealed the influence of HIV-related stigma on the transition journey of YPHIV, which provided a comprehensive understanding. The study limitation included selection bias, as we selected participants willing to join, which might represent the group with more positive experiences than those who did not show interest in participating. Although we conducted a multiple-method study and collected some quantitative data, the estimated sample size was to achieve qualitative data saturation and was probably too few to draw a conclusion or generalize to other young people living with HIV.*”
