# Supplementary material for: Understanding the challenges of healthcare transition in the context of HIV-related stigma for young adults with perinatal HIV in Thailand
Source: PLoS One. 2025 Nov 26;20(11):e0336624. doi: 10.1371/journal.pone.0336624 (PMC12654928; doi:10.1371/journal.pone.0336624)
Supplement: Attachment — Submitted filename: 20250828_Response to reviewers.docx [file pone.0336624.s004.docx]

**Response to reviewers’ comment**

1. Please provide additional background statistics on the two health facilities where the study was conducted, particularly in relation to the number of YPLHIV in care and key HIV indicators.

**Response:** We have added some information in the methods section to clarify background of the study population as follows.

*“We have similar cohorts of YPHIV who were initiated on HIV treatment and followed during 2005-2020 as a part of the TREAT Asia Pediatric HIV Observational Database [14]. In 2021, there were 124 and 145 YPHIV aged 18 years or older actively followed at the sites in Chiang Mai and Bangkok, respectively; their treatment outcomes during adolescent years were included in the previous publication [15].”*

2. Clarify whether PHQ-9 monitoring is part of routine HIV care at these sites and provide more detail on the relationship of caregivers to the YPLHIV interviewed.

**Response:** We added in the method that PHQ-9 is assessed as a part of regular clinic visits in both clinics.

“The most recent CD4 and viral load, self-reported adherence, and the Patient Health Questionnaire (PHQ-9) scores for depression, which were assessed on every clinic visit, were obtained from medical records.”

Details on relationships or caregivers were added in the footnote of Table 1 as follows.

*“Caregivers included eight biological mothers, one, biological father, and one stepmother.”*

3. Consider restructuring tables to separately describe (1) characteristics of the YPLHIV interviewed compared to the broader facility population, and (2) characteristics of caregivers.

**Response:** We would like to clarify that we did not have data on facility population that could be included in this report. As the study was designed to gather experiences from YPHIV during transitional period, data collection was focused on the qualitative data. Meanwhile, the characteristic table was only presented for readers to know the demographic background of informants.

4. Strengthen the opening of the discussion by defining low-level internalized stigma earlier and expand on whether Thailand has existing transition guidelines for YPLHIV or if these need to be developed—this will help better tie into the conclusions.

**Response:** We revised the first paragraph of the discussion as follows.

*“We found that more than half (60%) of YPHIV in this study had a low level of internalized stigma; the median stigma score was 14, which fell in the low range (< 16). Anticipated negative thoughts and negative thoughts toward self were common. Anticipated and enacted stigma, as well as discrimination experiences were revealed during IDI. Even though the national HIV treatment guideline has included the transition guidance, the outcomes remained unfavorable as evidenced in previous reports [7, 8, 15, 18]. Our study findings served as a complement to create understanding of the challenges faced by YPHIV.”*

5. Make the conclusion more robust by explicitly outlining the potential next steps, especially regarding training of healthcare workers involved in the transition of YPLHIV. Also include limitations as highlighted by the reviewer.

**Response:** We have revised the last paragraph of the discussion as follows.

*“The next steps in research might include gathering experiences from providers who are involved in the pre- and post-transition process to explore their perception and awareness of the current situation. Thorough understanding of the barriers and facilitators to effective transition would allow improvement of the process.”*

Also, we added the following statement in the conclusion

*“There is a need for practical and implementable healthcare transition guidelines or training for providers on the essential components including how to make individualized transition plans, counseling on management of internalized stigma, and follow-up strategies after transition.”*

6. Ensure consistency of style (e.g., use of reported speech) and correct minor omissions.
**Response:** Thank you. We have reviewed and reconciled the reported speech presentation as suggested.

Reviewers' comments:

7. I would recommend revising the 1st sentence in the introduction (line 53-54) to end...children born with perinatal HIV can grow to adulthood
**Response:** We revised the end of the sentence as suggested.

*“…can grow to adulthood.”*

8. Line 427 should be an opening statement of the discussion as it defines what Low level internalized stigma is and it should come before line 419.

**Response:** We revised the first two paragraphs of the discussion as suggested. The opening statement is now read as follows.

*“We found that more than half (60%) of YPHIV in this study had a low level of internalized stigma; the median stigma score was 14, which fell in the low range (< 16).”*

And we also revised the second paragraph of the discussion as follows.

*“The presence of HIV-related internalized stigma was not surprising. A larger study among 93 Nigerian youth with HIV (median age of 19.5 years) reported the presence of internalized stigma in 62% [18]. They found more negative self-image (stereotypes about HIV) in the younger age group (10-19 years) and more personalized stigma in the older age group (20-26 years). In our study, we enrolled selected participants who were willing to talk and share experiences; at the median age of 23 years, both components of internalized stigma were present. However, the study's low level of internalized stigma may not accurately reflect the broader population of YPHIV in the country, given their varied social contexts.”*

9. Line 431- I re-emphasize that we need to know how many YPHIV were on ARV treatment at these 2 health facilities in general.

**Response:** We added the details on study population in the Methods section as follows.

*“We have similar cohorts of YPHIV who were initiated on HIV treatment and followed during 2005-2020 as a part of the TREAT Asia Pediatric HIV Observational Database [14]. In 2021, there were 124 and 145 YPHIV aged 18 years or older actively followed at the sites in Chiang Mai and Bangkok, respectively; their treatment outcomes during adolescent years were included in the previous publication [15].”*

10. line 446- what proportion of YPHIV had care givers who were their parents...describe relationships of care givers to the YPHIV interviewed (this is related to the comment about a possible 2nd table focused on the care givers' characteristics.

**Response:** We added the relationship between interview caregivers and YLPHIV in the footnote of Table 1. We would like to clarify that most YLPHIV were orphaned and grew up with their relatives. However, most of caregivers who were still involved in HIV care and accompanied YLPHIV to clinics, which were the target population for this study, were biological parents or comparable (like a caring stepmother).


11. The Conclusion could be made more robust: not explicitly mentioned about actual plan for training of health care workers (especially those where YPHIV are being transferred to). FYI- It is unclear whether Thailand already has transition guidelines in place or these need to be developed. This should be mentioned in the discussion in order to tie up with the conclusion.

**Response:** We have revised the conclusion paragraph as follows.

*“The findings will be shared with HIV care policy makers, as there is a need for practical and implementable healthcare transition guidelines or training for providers on the essential components, including how to make individualized transition plans, counseling on management of internalized stigma, and follow-up strategies after transition.”*

We also mentioned the presence of Thailand’s guideline and the position of our study in the first paragraph of the discussion as follows.

*“Even though the national HIV treatment guideline has included the transition guidance, the outcomes remained unfavorable as evidenced in previous reports [7, 8, 15, 18]. Our study findings served as a complement to creating understanding of the challenges faced by YPHIV.”*

12. Statistical analysis: analytic methods took into consideration that this is a qualitative study and sample size is quite small to draw generalizable conclusions

**Response:** We have addressed the limitations in the last paragraph of the discussion as follows.

*“Although we conducted a multiple-method study and collected some quantitative data, the estimated sample size was to achieve qualitative data saturation and was probably too few to draw a conclusion or generalize to other young people living with HIV.”*

13. Language and presentation: For consistency in writing style, recommend using reported speech (line 365);

**Response:** We revised the description in the part as suggested. It is now read as follows.

*“Besides knowing about the transition plan, YPHIV also mentioned their need for HIV-related knowledge and wanted to learn more before moving out of the pediatric clinics. This requirement was reflected by a YPHIV who has transitioned to an adult clinic.”*

14. Line 510- Please correct .... counseling on management of internalized stigma (the word "of" is missing it seems)

**Response:** We corrected it. Thank you.

*“…management of internalized stigma,”*
