# Supplementary material for: Understanding the challenges of healthcare transition in the context of HIV-related stigma for young adults with perinatal HIV in Thailand
Source: PLoS One. 2025 Nov 26;20(11):e0336624. doi: 10.1371/journal.pone.0336624 (PMC12654928; doi:10.1371/journal.pone.0336624)
Supplement: Attachment — Submitted filename: 20251015 Response to reviewers.docx [file pone.0336624.s005.docx]

**Response to reviewers’ comments**

- Regarding data availability, we thank the reviewer for raising this important point. Due to ethical restrictions and participant confidentiality requirements approved by the Human Ethics Committee, the individual-level data cannot be made publicly available. However, de-identified aggregated data relevant to the study findings are presented in the supplemental files. More data is available upon reasonable request from the corresponding author at any time.

Reviewer #3: Please address these minor revisions:
1. Line 197: "..YA-PHIV.." should this be YPHIV?

**Response:** We corrected it. It is read as follows.

*“Not only did YPHIV have such fear(s) about HIV disclosure,…”*

2. Line 407-409: .."Whether with or without 407 neurocognitive impairment, their maturity may be lower than their chronological age. It limits their ability to comprehend and be capable of managing 408 health service-related tasks in the adult...". Is this a stated fact or speculation? If this is factual, please include your reference.
**Response:** This is both fact and speculation. We revised the sentence and added a reference as follows.

*“We found many of them were less mature than their chronological age. Whether with or without profound neurocognitive impairment, according to a meta-analysis, impaired cognitive function was reported in individuals with perinatal HIV when compared to uninfected population of similar age [18].”*

Line 410: "..mom.." would be more formal to use the word "mother" for this audience/publication

**Response:** Thank you, we changed from mom to mother as suggested.

*“A young man confessed that he still needed his mother…”*

Line 446: "..an US.." should this be "a US study"?

**Response:** We corrected it.
